# Progress of Veterinary Science

**Published:** 1885-07

**Authors:** 


### Progress of Veterinary Science.

Generative Organs of the Elephant.—The testes are small, globular, situated against the ilium, and suspended, each in its special tunica vaginates, freely in the abdomen below the posterior extremity of the kidney. The epididymis lies on the outer side, and the veins are remarkable for their number, large size and free communication. There are four prostrate glands. The free end of the penis is described as resembling a canal with a Y-shaped orifice. The female generative organs are in some respects very remarkable. The womb is almost wholly divided into two horns, and there is in the unimpregnated female a well-marked hymen. The vulva is very long, and so curved ordinararily that its loose external opening hangs down, and very like and in the position occupied in the male by the penis. It forms a regular prepuce to the clitoris, which in the young specimen examined by me was very like a small penis, and measured over a foot in length.—*Journal Veterinary Science in India.*

Castration of the Elephant.—Anatomical examination has shown that it is possible to reach the testes through an incision in the abdominal wall, but the operation must be performed on both sides. The huge bulk of the animal necessitates his being drawn into a hole dug in the ground after being thrown and properly secured. Then it is found that an incision in the usual position is of little use, for the animal, being "well ribbed up," the operator's arm is numbed by the severe compression it suffers between the last rib and the hip bone. An incision lower down is not of much value, because the testes is difficult to reach and the arm introduced through the wound is numbed by the pressure of the powerful abdominal muscles, as to prove to be unable to draw the testes down from its superior attachments, even if a fair grasp could be obtained (which is not generally the case). Thus it is evident that all attempts to perform this operation without chloroform and the instrument known as the ecraseur must be useless. Whether it can be done with these aids remains to be seen. Possibly division of the nerves of the penis might have the desired effect.—*Journal Veterinary Science in India.*

SCARLET FEVER IN CANARIES.—*British Medical Journal* reports cases of scarlet fever in canary birds, four of which were fed with bread mixed with the disquamations from a child having the disease. All four birds became ill; three recovered in two days, but the fourth, which ate with especial greed, was very sick, its skin being bright red, instead of the normally pale yellow color. It died. Dysphagic symptoms were observed.

THE FIRST AUTHENTIC CASE OF CONGENITAL TUBERCULOSIS.—For a long time it has been a question of earnest discussion whether intra-uterine, *i. e.*, congenital tuberculosis, ever occurred. For man, all the evidence thus far obtained, contradicts its occurrence in this form. Numerous cases of calves that seemed to indicate its possibility, but it has remained for our colleague and correspondent, Prof. Dr. Johne, of the Dresden Veterinary School, to give direct and authentic proof of the same. Johne is fast making a name in Europe as one of the leading pathologists, and ranks in Germany next to Koch in patho-mycology.

The case in point was a calf, eight months old, the mother of which was slaughtered, its lungs being found tuberculous to an excessive degree. Johne says, with regard to the microscopic examination of the diseased parts of the calf:

"The histological examination of the hardened tissues confirmed the existing views, so far as we are justified in speaking of a characteristic histological structure in tubercles, and the microscopical examination."

"All the nodes examined consisted of compact accumulations of round cellular elements of an epitheloid character. Towards the circumference of the noduli one could see numerous leucocytes, which became gradually less until they were entirely wanting in the healthy tissue enclosing the noduli. Numerous giant cells were present, and protoplesna-conglomerates in the large and very granular form, which is rather a peculiarity of bovine tuberculosis. The diseased parts were frequently calcified in places. The nodes were seated in the peri-bronchial tissues in the lungs, giving a typical picture of peri-bronchitis nodosa; in the liver they were to be seen in the interlobular tissue."

"The characteristic tubercle bacilli were well represented in the giant cells and in the tissues of individual noduli in the limits of the caseous elements, in the intermediate tissue before the caseous passed into the epitheliod elements of the tubercles. In the last-named elements no bacilli were to be found.

"The presence of the bacilli in the noduli proved their genuine tuberculous character, and that this case is the first known one of undoubted fœtal tuberculosis."

To the extinction of the bacilli Johne found the method recommended by Neelsen, and endorsed by Koch, superior to the ordinary aniline-oil combinations.

*Directions.*—1.00 (gramme one) of fuchsin is to be dissolved in 100.00 (grammes 100.) of an aquous 5 per cent. solution of carbolic acid, to which is to be added 10.00 (grammes 10.) of alcohol.

The tinction takes place rapidly and sharply. Sections of tissues are easily colored in 5 to 10 minutes.—*Fortschritte*, vol. 3, p. 198.

PERICARDITIS IN A HORSE.—Mr. Abraham read a paper on pericarditis in a horse, showing the specimen. The principle features were enormous hypertrophy and an extraordinarily extensive fibrinous exudation covering the whole pericardial surface. The normal weight of the horse's heart was six or seven pounds, but this specimen weighed twenty-one pounds. The notes of the case were taken by Mr. J. Kenny, V.S., under whose care the horse had been for pleuro-pneumonia, which had yielded to treatment. A week after the animal was brought back, with high pulse and friction sounds over the heart, subsequently becoming dull. At the *post-mortem* examination four gallons of yellow fluid was obtained from the pericardium. The hypertrophy of the heart was of long-standing and caused chiefly by the heavy work which the animal had to perform. The pericarditis appeared to be secondary to the pleuro-pneumonia. The immediate cause of death was the enormous pericardial effusion.

The President remarked that a chronic form of pericarditis, with effusion, had been observed by Cruveilhier, of Paris, who attributed it to over-work and the wearing out of the system. In the present case the question arose—Had the horse Bright's disease, and was the hypertrophy a result of sclerosis of the kidneys?

Mr. Collins said he had noticed in epidemics of influenza in horses that the disease principally attacked the serous membranes, and afterwards the pericardium. In 1856 the horses of the regiment to which he was attached at Manchester were decimated by an outbreak called influenza, and in every case the lungs and pleura were glued to the ribs.

Dr. Henry Kennedy remarked that there was in the museum a specimen of pericarditis in the cow.—*Dublin Medical and Surgical Journal.*

Foot and Mouth Disease Transmitted to Human Beings.—A German veterinarian constituted the existence of this disease in a herd of cattle on Jan. 9, 1883. Two days after he was called upon to ride quite a distance, the day being very cold and windy. He took the *same* handkerchief from his pocket to tie over his mouth that he had used on the day he inspected the cattle. In a few days he had a manifest attack of aphthous eruption, accompanied by fever and other constitutional disturbances. The attack lasted eight days. A 4-month's old child was fed with uncooked milk from a cow having the foot and mouth disease, and had an aphthous eruption in the mouth; two others suffered with acute diarrhœa, the disease lasting fourteen days. Two butchers caught the disease from slaughtering swine suffering therefrom.—*Fortschritte*, 1885, p. 301.

Contagious Pleuro-Pneumonia.—This disease is still reported as existing in Kentucky and Missouri, notwithstanding that the authorities supposed that it was entirely stamped out in the west, It must be distinctly understood that the fault does not lie so much with the Bureau of Animal Industry as with the Legislatures of the different States in which no appropriate laws exist, and where the ignorant doctrine of State rights comes into direct collusion with the efforts of the Washington authorities.

Unless more active measures be taken and successfully carried out than has as yet been the case this bovine pest will extend to the range cattle, and then we can say good-bye to all attempts at getting rid of it. We are doing our best to nationalize it at present.

Borax-Methylenblue as a Tinction in the Study of the Elements of the Central Nervous System, as well as the Coloring of

BACTERIA.—The addition of borax to a solution of methylenblue increases its coloring ability. The following have been found to be an appropriate mixture:

| | |
|---|---|
| Distilled waters | 40·00 |
| Saturated aquous solution of methylenblue | 24·00 |
| 5 per cent. borax solution | 16·00 |

Let it stand a few days and filter.

Sections to be placed in the above for from ten minutes to some hours, become dark blue in color; wash in wåter and alcohol until the grey substance becomes clear white, the white remains blue in color; treat *lege artis* and mount in Canada balsam. The latter must be softened with turpentine or benzole for all specimens colored with the anilines.

This preparation is only suitable for immediate examinations, as the color is not permanent.—*Fortschritte*, 1885, p. 280.

PREVENTIVE INOCULATION OF PLEURO-PNEUMONIA.—Certainly one of the most interesting historical and zoölogical facts recently unearthed by veterinary inquirers is one relating to the preventive inoculation of pleura-pneumonia by an African tribe. It is published in the *Annales de Médecine Véterinaire* of May, 1885. It appears that in 1880 the distinguished anthropologist and statician, Auatrefages, communicated to the Belgian Academy of Sciences the existence of a peculiar race of oxen in Senegambia, discovered by Dr. A. J. de Rochebrunne. This race, whose characters are fixed, is marked by a *third horn*, identical in its nature and mode of development with the frontal horns, but seated on the nasal region, where the bony elements, undergoing a sort of "functional osteoporosis" furnish a true core for it.

Now, it appears that since time immemorial, the Moors and other races of Senegambia have practiced preventive inoculation by plunging a knife or other instrument into the lungs of an animal, dead of epizootic pneumonia, and then "vaccinating" the herd on the nasal region. The development of the third horn at the spot of inoculation was supposed by Colin to be due in part at least to the local stimulation thus produced, but this theory is disregarded by the editors of the above-named journal. The latter conclude by saying: "The origin of the *bos triceros* will, without doubt, remain an enigma for a long time to come. But whatever it may turn out, the study of this race has made us acquainted with a process of preventive inoculation which indeed need not surprise us as being practiced by a people who from an equally distant period have been in the habit of inoculating healthy persons with the virus of those afflicted with small pox."

⁂

PROFESSOR BOULEY.—Some time ago a movement was started in Paris to commemorate the election of Prof. Bouley, the illustrious veterinarian, to the Presidency of the Académie des Sciences, of France—perhaps the highest scientific society or corporation in Europe—by presenting him with a gold medal of honor, specially struck for the occasion,—a replica of this in bronze to be given to each subscriber. This resolution having been duly notified to the veterinary profession, the call for subscriptions was readily responded to by veterinary surgeons in every part of the world, and the result was the collection of a sum ample for the oject in view. The medal was recently presented to M. Bouley by a deputation of veterinary surgeons, who waited upon him in Paris. The bronze copy has been forwarded to the subscribers, and from that now before us, there can be no doubt as to the appropriateness and artistic value of the medal, which has been designed by Roty. On the obverse is an excellent likeness of the first veterinarian who has occupied the exalted position of President of the Académie, and on the reverse is an allegorical group, designed to testify to the active and prominent part he has taken in introducing Pasteur's protective inoculation for certain deadly contagious diseases. This represents a human figure inoculating a sheep, and above is the motto. "ARTE NOVA PASTOR PECORUM CONTAGIA VINCIT, GRATUM OPUS AGRICOLIS." while words, "*Souvenir du* 26

*Janvier*, 1884," and the inscription, *Inspector-General des Eçoles Veterinaries, Commandeur de la Legion d'Honneur, Professeur au Museum—Les Eleves, Confrères, Les Amis*,"—the lower part having the figure of a coiled serpent, the head bent over a tablet on which it is inscribing. In the centre is a small space, in which the name of the subscriber is stamped. Altogether this souvenir of a great event is worthy of the occasion.—*Veterinary Journal.*

Cancer of the Penis, with Double Stricture of the Urethra and Disease of the Bladder.—J A. Miner, V.S., in the *Journal of Veterinary Science* in India, says a brown Australian gelding, about nine years old, was admitted in the hospital of the veterinary school, Lahore, on November 21st, 1884, suffering from what the owner described as warts on the penis, and had been treated for such by the salootree of a native cavalry regiment. The horse was very irritable, and it was impossible to examine the organ except when protruded in the act of passing urine. When admitted he was in very poor condition, and the coat was harsh and unthrifty; there was no difficulty in voiding urine, but it was passed frequently and in small quantities, about 2 to 4 ounces at a time, and was somewhat high-colored. The appetite was good. Attempts were made to examine the penis, but the horse was so irritable that they failed and, a complication, particularly about the bladder, was suspected. "The horse having shown slight pain on the 26th" the owner was communicated with, before casting the animal, and warned of the danger. *Carte blanche* having been given to do what was deemed necessary, the horse was thrown on the 28th; the penis was found to be in a very dirty state, as he would not allow it to be dressed, and when drawn out it was discovered that, with the exception of about three inches, the whole of the glands and free portion was extensively diseased. There was a fungus growth on the glands, distorting the urethral canal in a downward and backward direction. There were a considerable number of hard nodules imbedded in the substance of the organ, some of which had ulcerated on the surface. When they were cut into they were indurated, but had not undergone calcereous degeneration, nor were any of the kunkers usually present in bursattee sores discovered. The superficial inguinal glands were slightly enlarged. The diseased portion of the penis was removed, and the remaining part being so difficult to get hold of, considerable trouble was experienced in tying the arteries. While the patient was under chloroform the catheter was passed, some little obstruction being met with, and a few ounces of urine were drawn off, followed by a small quantity of mucus. A common dressing of sweet and encalyptus oil, 1 to 4, and, as there was some irritability, 4 drachms of chloral hydrate was given every morning in the form of an electuary, with formalation to the loins and enemata, the wound on the penis being dressed with the encalyptus oil by means of a pad of tow on a stick whenever possible, as he would not allow the part to be handled. Urine was passed in larger quantities, as mnch as a pint at a time, and not so frequently. He was again cast on December 4th, and the parts examined; they were healthy and doing well, except that a small nodule had formed on the surface of the wound, at the orifice of the urethral canal, but as it did not interfere with the passage of the urine it was decided to leave it alone. He was again cast on the 10th and 17th, and the wound appeared to be going on well, except that the orifice of the prepuce had become somewhat constricted: on December 19th, at 5 P.M., the horse was reported to be in pain, and the free flow of the urine obstructed, only coming away in drops. As it was then too dark to operate until next morning, fomentations were applied to the loins with anodyne enemata, and the chloral hydrate electuary given, but with little effect. The next morning, the 20th, the horse was thrown, and the nodule at the orifice of the urethra removed. An attempt was made to pass the catheter and a sound, but they failed to penetrate more than three inches up the canal. A soft metallic wire, doubled and twisted into a loop, was passed about eight inches, but was then stopped. The lining membrane of the urethra was drawn out and fastened by sutures backwards, and when released from the hobbles urine came away in drops. No relief was obtained, the animal got rapidly worse, and on the morning of the 21st, as he was evidently sinking fast and in very great pain, he was destroyed. The *post mortem* made next day revealed a stricture in the urethra, about three inches from the cut, and extending about four inches, and again, about four inches further up, a second one obstructing the urethra for about six inches; both of these strictures were so tight that it was with difficulty that a small probe could be passed. On opening the pelvis the bladder was seen to be partly distended with urine. It was emptied, and laid open along the long diameter from the neck; the urine that escaped was thick in consistence and tinged of a brown-red color. The inner surface throughout was coated with a greenish-purple slimy sediment, which floated away when washed, the walls of the bladder were abnormally thickened, and the mucous membrane discolored, showing several large clots of blood extravasation, and straining of the tissues. The neck of the bladder was somewhat thickened.

ANTHRAX.—[The following experiment was made by Fred. Smith to determine the incubative period of anthrax, and also to prove whether the virus of the disease could be conveyed by means of the digestive canal, and through an unbroken mucous surface. These points, as will be seen, were as thoroughly elucidated as one experiment can render them, and the case is placed on record owing to its important practical bearings.]

An adult horse received in two balls at 11.30 P.M. on the first day of the month a quantity of serum removed from the abdominal cavity of a fatal case of anthrax. The greatest care was exercised to roll these balls up securely to prevent them from breaking in the mouth, and they were rapidly and satisfactorily given by a very expert man, without any breaking having occurred. The following day (second) the morning temperature was 100.4 degrees, mid-day 100 degrees, and evening temperature 100 degrees; fed well all day. Third day, morning temperature 100 degrees, mid-day 1[illegible]0 degrees, evening 99.6 degrees; fed all day. Fourth day, temperature in morning 99.4 degrees, mid-day 100 degrees, evening 100.4 degrees. The temperatures were taken each day at 8 A.M., 12 noon and 5 P.M. At 5 P.M. on the fourth day the temperature was, as stated above, 100.4 degrees; at 7 P.M. (two hours afterwards) he was noticed to be uneasy, lying down, respirations very quick, *and the temperature* was now 107 degrees; at 8 P.M., temperature 107.2 degrees, at 9 P.M., temperature 106, exhibiting signs of colic, pulse very quick and small, trembling at the knees, respirations very much increased, in fact there was no doubt that the disease had thoroughly laid hold of the system. The temperature remained at 106 degrees until 3 A.M. the next day (fifth), and during this time all the symptoms became more aggravated; the fæces which passed were most offensive, flatus was continually passed, he constantly sat on his chest, and stretched out his head, resting on the chin, groaning, trying to roll, and before 3 A.M. the throat commenced to swell. At 4 A.M. he was standing, but still in pain, temperature 105.4 degrees; 5 A.M. still standing, appeared easier, throat more swollen, slight amber-colored discharge from nostrils; pulse can hardly be felt, coughs occasionally; 6 A.M. temperature 104.6 degrees; at 7 A.M. appeared much easier, temperature as before, 10 A.M. temperature 105, dyspnœa so great that I performed tracheotomy; noon, temperature 105 degrees, copious discharge from nostrils, throat much larger, and every symptom aggravated. Died very hard at 4.30 P.M., temperature 105 degrees.

The *autopsy* revealed extensive lung, but very few intestinal lesions, in fact, along the course of the lymphatics of the double colon (the most favorite site of anthrax œdema) there was but the least sign of disease, which would have escaped the eye of one not familiar with abdominal anthrax. There was no effusion beneath the kidneys or along the small bowels and mesentery, but there was acute gastro-enteritis of the mucous membrane.

The period of incubation in this case was about *sixty-seven hours*.

I at one time thought that the throat and lung forms of anthrax were due to ærial infection, and that the abdominal cases arose from infecton by the digestive canal. The experiment proves the error of this view. Here is a case of infection by the digestive canal causing acute throat and chest lesions, whilst some of the most significant intestinal changes were entirely absent. *—Journal Vet. Science in India.*

